# Ultra-Precise Dispensing for Rapid and Flexible Through-Silicon Via Filling

**DOI:** 10.3390/ma19091861

**Published:** 2026-05-01

**Authors:** Nina Szczotka, Shadi Nashashibi, Aleksandra Motyka, Sławomir Drozdek, Juerg Leuthold, Karol Malecha

**Affiliations:** 1 XTPL SA, Legnicka 48E, 54-202 Wrocław, Poland; aleksandra.motyka@xtpl.com (A.M.); slawomir.drozdek@xtpl.com (S.D.); 2 Department of Microsystems, Faculty of Electronics, Photonics and Microsystems, Wrocław University of Science and Technology, wyb. S. Wyspiańskiego 27, 50-370 Wrocław, Poland; karol.malecha@pwr.edu.pl; 3 Institute of Electromagnetic Fields (IEF), ETH Zurich, 8092 Zurich, Switzerland; shadi.nashashibi@ief.ee.ethz.ch (S.N.); leuthold@ethz.ch (J.L.); 4 Institute of Low Temperature and Structure Research, Polish Academy of Sciences, Okólna 2, 50-422 Wrocław, Poland

**Keywords:** through-silicon via, ultra-precise dispensing, additive manufacturing, conductive ink, metallization

## Abstract

**Highlights:**

- Use of Ultra-Precise Dispensing (UPD) for through-silicon via (TSV) filling.
- TSV aspect ratios of up to 14:1 with diameters down to 20 μm.
- Tailored silver-based nanopaste for high-aspect ratio filling.
- Flexible method for fast prototyping of TSV structures.

**Abstract:**

Three-dimensional integrated circuits (3D ICs) have emerged as a key technology to sustain scaling trends in the microelectronics industry. This advancement calls for a fundamental shift in how electrical interconnects are implemented, with through-silicon vias (TSVs) playing a pivotal role in enabling vertical connectivity between stacked chips. However, the metallization of TSVs traditionally involves elaborate and demanding processes, which can limit the speed and flexibility of prototyping and design modifications. In this paper, we investigate the use of Ultra-Precise Dispensing (UPD) technology of novel silver nanoparticle-based pastes as a simple and adaptable alternative to the metallization of TSVs process. The TSV filling process is outlined, followed by a detailed analysis of their morphology, filling quality, and electrical performance. We successfully achieve filled vias through a 280 μm thick silicon substrate with diameters down to 20 μm, resulting in an aspect ratio of up to 14:1, exhibiting favorable electrical properties. This work contributes to the achievement of dense, high-aspect ratio TSV fabrication using additive manufacturing, demonstrating a path towards reduced complexity of standard technology processes cycle, lower cost potential, and increased design flexibility.

## 1. Introduction

The continuous development towards smaller, faster, and more power-efficient electronic and electrooptic devices has made three-dimensional (3D) integrated circuit (IC) packaging a critical area of research and development [1,2,3,4]. At the core of 3D integration lies the through-silicon via (TSV), a vertical interconnect that passes through the silicon substrate, enabling the stacking and interconnection of multiple dies [5,6,7,8]. TSVs serve as crucial pathways for transmitting signals and power between vertically stacked chips, offering significant benefits over traditional wire bonding and flip-chip technologies, including shorter interconnect lengths, reduced signal latency, lower power consumption, and higher input/output (I/O) densities [9]. Typical TSVs feature micrometer-scale dimensions, with diameters ranging from approximately 5 μm to tens of micrometers and depths spanning from tens to hundreds of micrometers, with a commonly reported aspect ratio of up to 15:1 [10,11,12,13,14]. The ability to create dense vertical interconnections has opened up new possibilities for heterogeneous integration, allowing the combination of dies with different functionalities and fabrication processes within a single package and play a critical role in the future of microelectronics [15].

While electroplating has been an established technique for metallizing through-silicon vias (TSVs), valued for its ability to yield highly conductive bulk copper, it requires a complex and multi-step fabrication pathway [16]. This method typically mandates a conductive seed layer, the electrochemical deposition process itself, and subsequent removal of resist and excess material (overburden) often via Chemical–Mechanical Polishing (CMP) (Figure S1). Such a process is inherently time-consuming and can be particularly challenging for achieving defect-free filling in high-aspect-ratio TSVs, where issues like void formation, seam creation, and limitations in mass transport of plating additives are common [17]. Furthermore, its reliance on an electric field restricts its applicability to conductive substrates or substrates with a pre-deposited conductive layer. For research and development, electroplating’s complex, interdependent steps make it less flexible and more difficult to quickly modify or optimize for novel designs and rapid prototyping.

In the pursuit of advanced and efficient electronics and packaging, Additive Manufacturing (AM) has emerged as a promising alternative for direct material deposition, offering high precision and reduced processing complexity [18,19,20,21]. Unlike conventional TSV metallization methods, AM processes can provide conformity to complex 3D topographies and address limitations in prototyping, sparking interest in the microelectronics packaging industry. Various additive techniques are being explored for TSV metallization and related applications. For example, inkjet (IJ) printing is a relatively mature AM process that shows promise in filling hollow structures with dielectric polymers and depositing metallic layers with precise droplet control [22]. However, it faces challenges such as inferior feature sizes, poor metallic ink conductivity, the coffee-ring effect, and overspray. Electrohydrodynamic (EHD) printing can achieve finer resolutions by controlling extremely small droplet volumes, but it requires an electric field which can be detrimental to fragile electrical components.

Ultra-Precise Dispensing (UPD) arises as a promising AM alternative, representing a compelling paradigm shift for TSV manufacturing and packaging [23]. As a pressure-driven, direct writing method, UPD precisely extrudes high-viscosity conductive pastes (such as silver nanoparticle-based inks with high solid content) through microscale nozzles (0.5–10 µm). UPD enables the substrate agnostic creation of fine features (down to 1 µm) with high aspect ratios and complex 3D topographies without the need for an electric field. Furthermore, the range of conductive and insulating materials available with UPD significantly distinguishes the technology from electroplating. While copper, used in electroplating, demands extensive post-deposition cleaning, protective agents against oxidation, and inert gas for sintering, the silver nanoparticle-based inks in UPD simplifies the workflow [4]. Sintering of dedicated silver inks can occur in ambient air under Complementary Metal–Oxide Semiconductor (CMOS) compatible conditions, and it does not require additional protective layers, offering simpler material handling and processing advantages for 3D microelectronics [24]. The benefits of UPD provides substantial flexibility for the Research and Development departments, making it highly adaptable for rapid prototyping and developing custom demonstrators where process variables can be adjusted with relative ease. A comprehensive comparison of these additive approaches, including UPD, with traditional electroplating methods is provided in Table 1.

This study explores the application of UPD using an advanced silver nanoparticle paste for TSV filling, focusing on achieving high-resolution printing and high aspect ratio filling with aspect ratios of up to 14:1 (280 µm:20 µm). We investigate the structural changes within the silver paste during the critical sintering process, a fundamental step for achieving the desired electrical properties. By analyzing these phenomena, this work aims to provide comprehensive insights into UPD’s suitability as a reliable TSV filling technique. A notable aspect highlighted is the inherent simplicity, as this method avoids complex fabrication steps and post-processing. The findings contribute significantly to the field of a new generation of TSV filling strategies for advanced packaging, underscoring UPD’s potential as a versatile tool in microelectronics back-end-of-line integration.

## 2. Materials and Methods

### 2.1. TSV Filling Process

**Figure 1:**
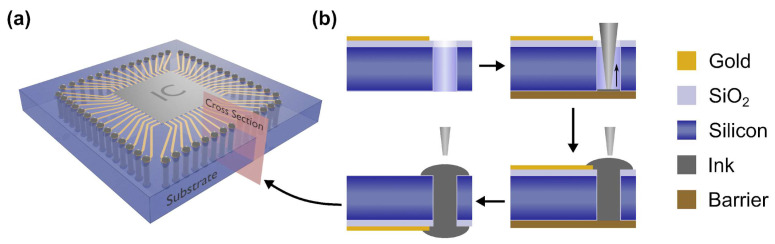
(**a**) Illustration of TSVs connecting an integrated circuit with the backside of the chip for advanced 3D packaging. (**b**) Process steps for the TSV filling. TSV holes with gold connections are fully filled from the frontside. The chip is then flipped, and the via is filled from the backside, with the option of creating additional conductive tracks with the conductive ink.

Our vision is to simplify and broaden TSV metallization by moving away from conventional complex processes. We demonstrate that Ultra-Precise Dispensing of silver nanoparticle-based pastes offers a versatile alternative for reliably filling TSVs as envisioned in Figure 1a. Samples were prepared as outlined in “Section 2.2. Chip Fabrication” and resulted in chips with TSV holes and gold connections for the electrical characterization test structures. An XTPL Delta UPD system (XTPL SA, Wrocław, Poland) was utilized for filling the TSVs holes with a conductive silver paste. Nozzle diameters were selected to ensure at least 5 µm clearance from the TSV walls, ranging from 5 µm up to 40 µm for larger TSVs to optimize deposition speed.

The overall fabrication strategy involved multi-step processing, as shown in Figure 1b: Initially, the TSVs were filled from the top side of the wafer and sintered. Subsequently, the substrate was physically inverted, the sacrificial barrier layer removed, and interconnects were then fabricated from the bottom side of the chip. This approach allowed for the creation of integrated structures, such as daisy chains, by connecting multiple filled TSVs in series from the bottom side, in conjunction with the gold electrodes on the top surface.

The automated filling process involved precisely positioning the nozzle directly above a TSV, then lowering it into the via until the nozzle tip made contact with the bottom barrier layer. Material deposition commenced at the bottom, with the nozzle simultaneously dispensing paste and slowly retracting upwards at a controlled speed to ensure continuous filling of the via. Upon reaching the top edge of the hole, material deposition ceased, and any accumulated material on the nozzle formed a precise “bump” on the surface of the filled via. This bump was intentionally created as part of this single-pass filling process, where additional paste was applied upon nozzle retraction and cessation of dispensing, forming a precise feature on the via’s top opening. This process effectively combines via filling and bump creation into one continuous step. These bumps facilitated the electrical contact with adjacent features, such as the pre-existing gold collars on the substrate surface. The nozzle then lifted to a predetermined height before moving to the next TSV, allowing for sequential and automated filling of multiple holes. Following deposition, the filled TSVs underwent thermal sintering on a hotplate at 200 °C for 10 min in an ambient air environment. Solvent evaporation from the silver paste occurred during this heating process. While the systematic optimization of the paste’s chemical formulation constitutes a separate, proprietary developmental effort, the dispensing process was rigorously optimized to achieve precise and defect-free deposition. This optimization involved fine-tuning pressure levels for material dispensing at the bottom of the via, during upward movement, and adjusting the speeds for both downward movement into the via and upward retraction during deposition. Specific optimized values for pressure were in the range of 5–7 bar to ensure the complete volumetric extrusion of the high-viscosity paste. However, the upward retraction speed emerged as the most critical variable. Extensive experimental iterations were conducted to establish an optimal retraction speed = 0.07 mm/s that precisely balanced the required filling volume with the overarching goal of maximizing process throughput. Additionally, the tool’s trajectory control was optimized to enable fully automated, seamless transitions between adjacent TSVs across the array, minimizing non-dispensing idle time. Ambient temperature and humidity conditions during the deposition process were maintained within the operational limits of the UPD printer. Each TSV was filled individually and completely in a single pass; there were no repeated downward passes or delays between filling individual vias.

### 2.2. Chip Fabrication

Intrinsic <100> silicon wafers with a thickness of 280 µm were used as a starting point for sample preparation. A SiO_2_ layer of 150 nm was deposited for insulation of the silicon substrate using Plasma-Enhanced Chemical Vapor Deposition (PECVD). Gold connections were fabricated on top of the SiO_2_ layer using photolithography and E-Beam Evaporation (EBE) of 5 nm Ti/100 nm Au followed by a Lift-Off Process (LOP). The TSV holes were prepared using photolithography, Reactive Ion Etching (RIE), and Deep Reactive Ion Etching (DRIE). A 30 nm SiO_2_ layer was then deposited by Atomic Layer Deposition (ALD) for insulation within the TSVs. Finally, SiO_2_ covered gold contact pads were made accessible by photolithography and a subsequent RIE step. Test samples included various chips with different TSV diameters ranging from 20 to 200 µm, varying pitches (typically 150 to 600 µm between circular TSVs), and a diverse number of in-series connected TSVs to allow for comprehensive electrical characterization. A schematic process flow can be found in Supplementary Figure S4.

### 2.3. Silver Paste

Various silver nanoparticle-based pastes were employed for filling the TSVs. One primary paste utilized was CL85, which consisted of spherical Ag nanocrystals with an average diameter of 30–50 nm and 83 wt.% metal content, and exhibited a high viscosity of 1–2 million cP at a shear rate of 0.2 s^−1^. In addition, a series of custom-formulated RnD pastes (RnD_1, RnD_3, and RnD_4) were also employed. Notably, these pastes exhibited excellent long-term stability, allowed for dispensing in the form of thin lines, and demonstrated good adhesion to both glass and silicon substrates. 

### 2.4. Filling Preparation

Prior to TSV filling material deposition, the wafers were cleaned with isopropanol. To ensure precise filling and prevent uncontrolled material outflow into unwanted areas at the bottom of the vias, a sacrificial barrier layer in the form of Kapton type was applied before the filling process; this barrier was subsequently removed manually after the deposition process to enable through-wafer electrical connections.

### 2.5. Characterization

For characterization of the filled structures and observed deformation, an Olympus LEXT OLS5100 Laser Confocal Microscope (Evident Corporation, Tokyo, Japan) and Helios NanoLab 600i Scanning Electron Microscopy (SEM) (Thermo Fisher Scientific, Hillsboro, OR, USA) with a Schottky gun and FEI Helios G4 CXe Plasma Focused Ion Beam (PFIB) CXe cross-sectioning (Thermo Fisher Scientific, Hillsboro, OR, USA) were employed. Electrical resistance measurements were also performed using the two-point probe method with a Keysight 34461A Digital Multimeter (Keysight Technologies, Santa Rosa, CA, USA) on the fabricated interconnects to assess their resistance, utilizing daisy chain structures for characterization (Figure S2).

## 3. Results and Discussion

### 3.1. Morphological Characterization

**Figure 2:**
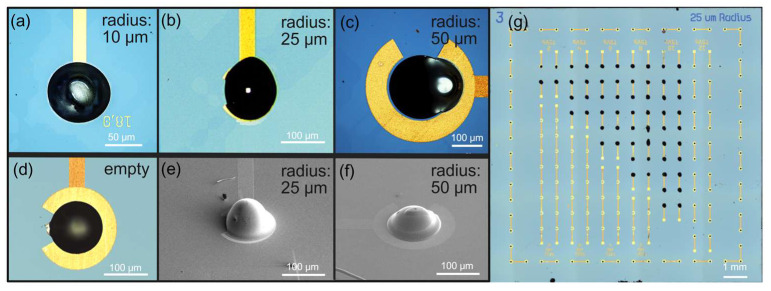
The surface images of the silicon substrate with filled and empty vias were captured using confocal microscopy (**a**–**d**,**g**) and Scanning Electron Microscopy (SEM) (**e**,**f**). (**a**–**c**) Confocal images of vias filled with silver RnD_4 paste using UPD technology, shown on substrates with different TSV radii: 10 µm, 25 µm, and 50 µm, respectively. A distinct bump was intentionally created on the top of each via to provide a proper electrical connection to the adjacent gold collar. (**d**) Confocal image of the top view of an empty via with a 50 µm radius, provided for visual comparison. (**e**,**f**) SEM images of a filled via and its connection to the traces of the gold collar, presented from a tilted perspective to highlight the three-dimensional morphology of the bump. No visual defects were observed in the global top-down view of the samples. (**g**) Confocal image displaying a representative daisy chain configuration, which illustrates the path of the current from the top side. The distance between subsequent structures in the chain is 560 µm. From this top view, the vias appear fully filled and functional.

Morphological characterization was performed using confocal microscopy and SEM to assess the quality of the TSV filling and the surface morphology of the printed structures (Figure S3). Top-view (Figure 2a–c) observations consistently revealed uniform filling across the vias. The surface of the filled areas appeared smooth, with no noticeable roughness or irregularities. A unique and deliberate characteristic observed from the top view was the presence of a distinct bump on the surface of each filled via. This key difference will be clearly highlighted by contrasting the appearance of an empty via (Figure 2d) with a fully filled via prominently displaying the uniquely formed bump on its surface. No apparent defects or cracks were observed on the top surface of the filled vias or the surrounding printed features (e.g., gold electrodes). This was further confirmed by tilted SEM images (Figure 2e,f), which provided a three-dimensional perspective of the filled via and the gold collar traces. Figure 2g displays the substrate’s surface, revealing the representative daisy chain configuration, where vias are spaced 560 µm apart. A functional connection to another material or substrate is enabled by a frame of vias created around the entire sample.

### 3.2. Cross-Section Inspection

**Figure 3:**
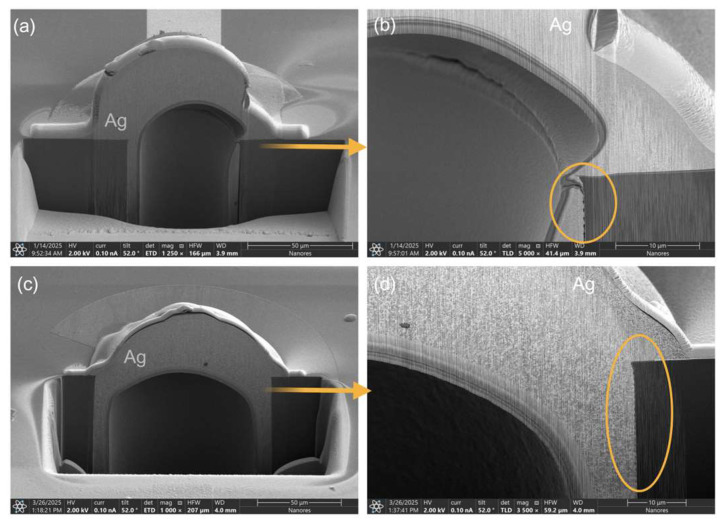
Cross-sectional SEM images illustrate the filling quality of TSVs fabricated with two distinct silver nanoparticle pastes. Panels (**a**,**c**) show the overall cross-sectional morphology, while panels (**b**,**d**) provide magnified views of the critical via wall interface. Panels (**a**,**b**) depict a TSV filled with “CL85” paste, showing inconsistent adhesion and insufficient filling at the corner of the right sidewall, a particularly sensitive area. In contrast, panels (**c**,**d**) show a TSV filled with “RnD_4” paste, where the material is distributed uniformly and adheres well to both sidewalls, effectively filling these critical regions. The hollow center, consistent with the described sintering mechanism, is visible in all filled vias.

Cross-sectional observations using SEM-PFIB revealed the internal morphology and filling quality of the TSVs. Post-sintering cross-sections consistently demonstrated internal voids within the filled TSVs, typically occupying approximately half of the cross-sectional area (Figure 3). The assessment of filling quality in the bottom sections, however, was constrained by analytical limitations. Despite the presence of voids, the silver material exhibited consistent adherence to the TSV walls. Deformation, manifesting as material extrusion from the via opening, occurred during the sintering process. This phenomenon is attributed to the evaporation of organic components and subsequent material shrinkage, which leads to internal gas evolution and material displacement within the confined via. The morphological characteristics, particularly wall adhesion, were influenced by the paste formulation. The commercially available universal version “CL85” paste exhibited reduced wall adherence (Figure 3a,b), whereas successive modifications to the “RnD” pastes progressively enhanced wall contact (Figure 3c,d), demonstrating their crucial specialized use in this process. Importantly, TSV diameter, depth, aspect ratio, sintering parameters, and printing speed demonstrated no significant influence on overall filling quality or deformation within the tested range. More than 80% of fabricated vias exhibited electrical conductivity. The remaining instances, where electrical flow was not established, are attributed to open circuits formed during the early process calibration phase; the precise mechanisms governing these failures are thoroughly analyzed and discussed in “Section 3.5. Subsequent investigations” will focus on elucidating the precise modification of the surface bump and characterizing the material behavior at the critical silicon-silver interface near the top of the via.

### 3.3. Electrical Characterization

**Figure 4:**
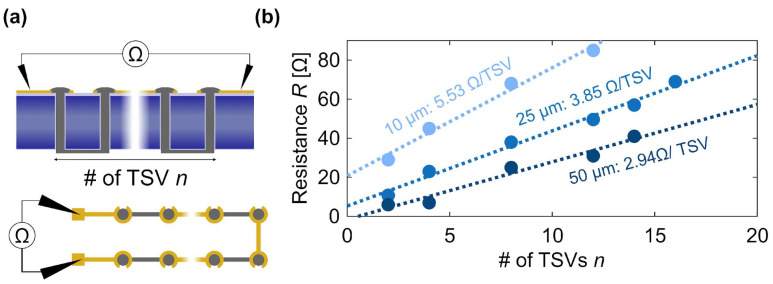
(**a**) Cross-sectional view (**top**) and top view (**bottom**) of the characterization method. The resistance of daisy chains with varying numbers of TSVs—alternatingly connected on the front side (gold traces) and back side (printed silver traces) of the sample—is measured using electrical probes. # - number (**b**) Resistance values of daisy chains of different lengths and TSV radii (10 μm, 25 μm, and 50 μm) through a 280 μm thick silicon substrate (measurements are based on two samples per condition - N = 2). The dotted lines represent linear fits to the data, with their slopes directly yielding the total resistance per TSV R_TSV,total_. It is important to note that the extracted resistance values represent an upper limit for the individual TSVs. The two-point measurement setup inherently encompasses the resistance of the top gold contact pads and the backside printed silver traces. Specifically, the backside traces exhibited an average resistance of 1.15 Ω (measured across N = 16 trace segments). Consequently, the intrinsic resistance of a single UPD-filled silver TSV is expected to be lower than the extracted TSV R_TSV,total_ (#-number).

To assess the resistance per filled TSV, a daisy chain test structure with in-series connected TSVs was fabricated and characterized. Filled TSVs were connected by gold connections on the front side and by printed silver traces on the backside, as shown in Figure 4a. Having a varying number of TSVs connected in series allows for the precise measurement of resistances by minimizing the effect of contact resistance of the used probes. TSVs with radii of 10 μm, 25 μm, and 50 μm were daisy-chained with varying amounts of vias and the resistance values measured as shown in Figure 4b using a two-probe system. The resistance follows a linear trend for all the radii and extracting the slope with the help of a linear regression provides resistance values *R*_TSV,total_ of 5.53 Ω TSV^−1^, 3.85 Ω TSV^−1^, and 2.94 Ω TSV^−1^ for 10 μm, 25 μm, and 50 μm radius, respectively. These extracted resistance values per TSV *R*_TSV,tot_ include the TSV resistance R_TSV_, the resistance of the gold/silver traces connecting neighboring vias *R*_TSV,trace_ as well as the contact resistances between the gold connections and the silver paste used for TSV filling *R*_TSV,contact_. Using resistivity values of gold and silver and the dimensions of the traces, an average trace resistance per TSV *R*_TSV,trace_ of roughly 4.9 Ω TSV^−1^, 2.5 Ω TSV^−1^, and 1.9 Ω TSV^−1^ is estimated for 10 μm, 25 μm, and 50 μm radius, respectively. These calculations of the trace resistances suggest that they dominate the total resistance per TSV. The measured values provided in this paper therefore represent an upper limit for the resistance of the TSV alone, which has an estimated resistance R_TSV_ in the range of 1 Ω. While these extracted values highlight representative measurements for each geometric variant (N = 2 per specific TSV radius), they build upon a broader preliminary testing phase involving dozens of functional vias to successfully validate this proof-of-concept.

To contextualize these electrical characteristics, a standard copper electroplated TSV of comparable dimensions (e.g., 20 µm diameter, 100–200 µm depth) typically exhibits a resistance in the range from 5 to 50 mΩ [2,28]. The higher resistance of the UPD-sintered silver vias (~1 Ω) is primarily attributed to the inherent porosity of the sintered nanopaste and the absence of bulk metal density. However, this electrical performance should be evaluated against the reduction in process complexity. As illustrated in Supplementary Figure S1, conventional Cu metallization involves vacuum seed layer deposition, electroplating, and subsequent CMP. Achieving defect-free filling in high-aspect-ratio structures via electroplating typically requires 3 to 12 h, depending on the additive composition and external assistance [2,30]. In comparison, the UPD method enables the complete volumetric filling of a single high-aspect-ratio TSV in approximately 20 s. Therefore, while the absolute via resistance is higher, the elimination of CMP and seed layer processes, coupled with the rapid deposition rate, establishes UPD as a highly viable technological trade-off for rapid prototyping and agile back-end-of-line applications where ultra-low via resistance is not the primary constraint.

### 3.4. Modern Material Modification

Copper has been the standard material for via filling due to its high electrical and thermal conductivity. However, its thermal expansion mismatch with silicon induces stress and piezoresistive effects, affecting electrical performance and reliability [22,31]. Copper TSVs also complicate wafer thinning and increase the risk of warping or cracking, particularly in ultra-thin DRAM stacks [6].

To address the demands of advanced TSV applications, materials based on silver nanoparticles are being explored. This approach provides several advantages, including low sintering temperature, compatibility with additive manufacturing, high electrical conductivity, and potential for fine-pitch miniaturization. The tested materials, designated as “RnD_1”, “RnD_3”, and “RnD_4”, were developed as specific modifications of the standard “CL85” silver nanoparticle-based paste. These modifications were driven by the need for materials specifically suited for dispensing into high-aspect ratio structures and designed to compensate for the material shrinkage that occurs due to the evaporation of organic components during the sintering process. The “RnD” pastes were meticulously adjusted to meet these application-specific requirements, simplifying their composition to a minimum to prevent contamination and solvent evaporation problems. All three pastes have a high metal content and a tendency to thin when properly sheared, which is critical for their dispensing performance. Specifically, “RnD_1” and “RnD_3” feature new polymers, dispersing agents, and a liquid rheological additive based on polyamide. The “RnD_4” sample, in turn, features an unusual combination of solvents and dispersing additives, which ensures the paste’s unique properties during dispensing into vias. Their detailed properties and characteristics are summarized in Table 2.

### 3.5. Extrusion

Our results demonstrate a deformation of the silver nanoparticle-based paste within the TSVs during the sintering process, a phenomenon attributed to thermomechanical stresses. These stresses arise from the significant Coefficient of Thermal Expansion (CTE) mismatch between the silver nanoparticles-based paste and the silicon substrate (αSi = 2.8 (300 K) − 3.1 ppm K^−1^ (423 K)) [32]. The literature indicates that the CTE for silver nanoparticles and pastes varies considerably, typically ranging from approximately 6 ppm K^−1^ in vacuum up to 75 ppm K^−1^ or even higher in air or within a matrix. Given this range, the silver paste’s CTE is likely substantially different from that of silicon.

**Figure 5:**
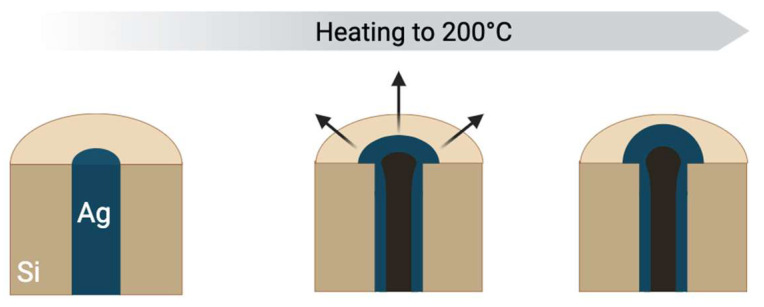
The stages of extrusion formation with an increase in the sintering temperature (Direction: expansion of paste).

As the temperature increases during sintering, this CTE mismatch induces substantial thermomechanical stresses [16]. Within the confined TSV structure, which is constrained in the x, y, and z directions except for the top opening, these stresses are exacerbated. The silver paste’s inability to expand laterally or downwards under high thermal loads compels the material to expand upwards. This directional expansion leads to plastic deformation of the silver material at the top of the TSV, resulting in the observed deformation, analogous to the extrusion phenomena reported for copper in TSVs [6]. Furthermore, continued temperature increase and plastic deformation can also lead to the formation of internal voids or empty spaces within the bulk of the filling material. The evolution of this deformation process with the increase in temperature will be further illustrated in Figure 5. This highlights the critical importance of comprehensively considering both the CTE mismatch and the structural constraints during the design and processing of reliable TSVs with silver nanoparticle-based filling materials.

While the CTE mismatch drives the upward extrusion, the formation of internal voids within the UPD-filled vias is governed by a fundamentally different mechanism than those found in conventional copper electroplating. In electroplated Cu TSVs, especially at high aspect ratios, voids (often referred to as seams or keyholes) typically form due to mass transport limitations; the plating solution becomes restricted, trapping chemicals or air as the via closes from the top down [28]. In contrast, the voids observed in our UPD process originate primarily from volumetric shrinkage. As detailed in Table 2, the custom silver nanoparticle pastes contain approximately 78 wt.% metal, with the remaining 22 wt.% consisting of organic components such as solvents, polymers, and dispersing agents. During the 200 °C sintering stage, the removal of organic phase occurs sequentially, involving the primary evaporation of the solvent and the subsequent thermal decomposition of the polymeric stabilizing agents. Because the rigid confinement of the via walls prevents lateral compensation, this significant solvent evaporation inevitably leaves behind internal empty spaces alongside the upward material extrusion.

Furthermore, this physical mechanism directly explains the approximately 80% electrical yield observed in this study. Because this work serves as an early-stage proof-of-concept for the UPD method conducted on an actively evolving process, the printing parameters were continuously calibrated to maximize throughput. Specifically, we tested the critical balance between maintaining a high dispensing pressure (up to 10 bar) and varying the upward retraction speed to achieve the rapid 20-second filling time. In specific test instances where the retraction speed was set too high relative to the applied pressure, an insufficient volume of wet paste was deposited into the via. After the subsequent organic mass loss during sintering, this volumetric deficit resulted in physical gaps within the silver path, creating open circuits (non-conductive vias). Therefore, the ~20% failure rate represents the necessary sacrifices made during this initial, dynamic calibration phase to pinpoint the optimal process window. With the structural mechanics and parameter dependencies now thoroughly understood, ongoing automated optimizations dynamically adjust the dispensing speed and pressure to deposit a calculated excess of material. This strategy actively pre-compensates for the sintering-induced shrinkage, preventing open circuits and driving the process yield toward 100% in future high-volume iterations.

## 4. Conclusions

This study investigated the use of UPD with modern silver nanoparticle pastes for high-resolution and high-aspect-ratio TSV filling, achieving aspect ratios of up to 14:1. Morphological analysis revealed uniform top-surface fills with distinct contact bumps. The vias demonstrated electrical conductivity with measured resistances of 5.53 Ω TSV^−1^ (10 µm radius), 3.85 Ω TSV^−1^ (25 µm radius), and 2.94 Ω TSV^−1^ (50 µm radius) through a 280 µm silicon substrate. These values provide an upper limit for via resistance due to parasitic resistances. Post-sintering observations indicated internal voids and material extrusion, attributed to thermomechanical stresses from CTE mismatch and volatile evaporation. To address these challenges, future work will focus on specific steps to mitigate void formation, improve interfacial adhesion, and strictly control material extrusion during the sintering phase. This will involve establishing key criteria for next-generation pastes to achieve even higher electrical conductivity and structural reliability. Furthermore, while this study establishes the fundamental feasibility of UPD for via filling, we acknowledge that long-term reliability is absolutely essential for validating TSV performance in real-world applications [33,34,35]. Therefore, subsequent research phases are planned to include comprehensive thermal cycling tests (TCTs) and electromigration (EM) stress assessments to rigorously evaluate the electro-thermomechanical stability of these silver nanoparticle TSVs. Additionally, to transition this technology toward high-volume manufacturing, future efforts will explore scalability modifications and optimized dispensing strategies to significantly increase production throughput. The UPD workflow streamlines the fabrication process by offering simplified post-processing and reducing the overall number of required steps for TSV preparation. This research highlights UPD’s potential as a versatile technology for reliable TSV filling, representing a new generation and approach to TSV fabrication in advanced microelectronics.

## Figures and Tables

**Table 1:** A comprehensive comparison of conventional electroplating, inkjet printing, and UPD for TSV filling, highlighting key differences in process complexity, material properties, and fabrication capabilities.

Feature	Conventional Electroplating (e.g., Copper) [5,6,25,26]	Inkjet Printing [22]	Ultra-Precise Dispensing (UPD) [27]
**Method Type**	Electrochemical deposition (often multi-step)	Additive manufacturing (drop-on-demand)	Additive manufacturing (direct ink writing)
**Process Complexity**	Multi-step (seed layer, resist, plating, removal, CMP)	Simplified (direct deposition, fewer steps)	Simplified (direct deposition, fewer steps)
**Electric Field Requirement**	Required	Not required	Not required
**Substrate Compatibility**	Requires conductive seed layer	Compatible with various substrates	Compatible with various substrates
**Process Concerns**	Limitation when scaling to higher aspect ratios [28]	Overspray, lower resolution [29]	Potential for minimization via controlled deposition (e.g., high-viscosity paste filling)
**Material Overburden**	Common, requires Chemical–Mechanical Polishing (CMP)	Reduced, targeted deposition minimizes waste	Minimal/Localized, reduced material waste
**Processing Time**	Time-consuming for deep vias	Variable, potentially efficient for targeted filling	Potentially reduced for direct filling
**Material Form**	Ions in electrolyte (low viscosity)	Low-viscosity inks (strict rheological requirements)	High-viscosity pastes (e.g., up to 90 wt.% solid content)
**Feature Resolution**	Defined by lithography and plating capabilities	Coarse (e.g., 10–40 um)	Fine features (down to 1 um)
**Typical Conductivity**	High (approaches bulk copper conductivity)	Can be poor for metallic inks (limited by organic residue)	Varies, can be lower than bulk metal (e.g., up to 45% of bulk silver for sintered paste)
**Reliability Concerns**	Thermomechanical stress, void-related issues	Coffee-ring effect, strict rheological requirements, organic residue, general reliability concerns	Material shrinkage during sintering, long-term reliability requires investigation
**Cost**	High equipment and process complexity cost	Potentially lower equipment and tooling costs (maskless, digital)	Potentially lower equipment and operational costs for small-scale/R&D
**R&D Flexibility**	Limited adaptability—complex, interdependent steps	High flexibility—digital, maskless process enables rapid prototyping	High flexibility—precise control and versatile materials enable prototyping

**Table 2:** Summary of the key properties and characteristics of the silver nanoparticle-based pastes (“CL85”, “RnD_1”, “RnD_3”, and “RnD_4”) used for TSV filling.

Paste Name	Nanoparticle Size [nm]	Metal Content [wt.%]	Viscosity [cP]	Shear Rate [s^−1^]	Rheological Additive/Components	Special Characteristics
**CL 85**	30–50	83	1–2 million	0.2	-	Spherical Ag nanocrystals
**RnD_1**	35–50	76	Several hundred thousand	0.2	Polymer, dispersing agent, polyamide-based liquid	Characteristic rheological profile; tendency to dilute with shear
**RnD_3**	35–50	78	Several hundred thousand	0.2	Polymer, dispersing agent, polyamide-based liquid	Characteristic rheological profile; tendency to dilute with shear
**RnD_4**	35–50	Similar to others	Similar to others	-	-	Unusual solvent combination for microbead dispensing

## Data Availability

The data presented in this study are available upon request from the corresponding author due to intellectual property restrictions and proprietary company information.

## Supplementary Materials

The following supporting information can be downloaded at: https://www.mdpi.com/article/10.3390/ma19091861/s1. Figure S1: Scheme of common via filling process using electroplating; Figure S2: Electrical resistance measurement setup; Figure S3: SEM images of filled vias; Figure S4: Full process flow from silicon substrates to filled vias.

## Abbreviations

The following abbreviations are used in this manuscript:

TSV	Through-Silicon Via
UPD	Ultra-Precise Dispensing
3D ICs	Three-Dimensional Integrated Circuits
CMP	Chemical–Mechanical Polishing
AM	Additive Manufacturing
IJ	Inkjet
EHD	Electrohydrodynamic
CMOS	Complementary Metal–Oxide Semiconductor
PECVD	Plasma-Enhanced Chemical Vapor Deposition
EBE	E-Beam Evaporation
LOP	Lift-Off Process
RIE	Reactive Ion Etching
DRIE	Deep Reactive Ion Etching
ALD	Atomic Layer Deposition
SEM	Scanning Electron Microscopy
PFIB	Plasma Focused Ion Beam
CTE	Coefficient of Thermal Expansion
TCT	Thermal Cycling Tests
EM	Electromigration
