# Supplementary material for: Ultra-Precise Dispensing for Rapid and Flexible Through-Silicon Via Filling
Source: Materials (Basel). 2026 May 1;19(9):1861. doi: 10.3390/ma19091861 (PMC13165430; doi:10.3390/ma19091861)
Supplement: Supplementary file 1 [file materials-19-01861-s001.zip › materials-4248188-Supplementary.pdf]

materials

Supplementary Materials

Ultra-Precise Dispensing for Rapid and Flexible Through-Silicon via Filling

Nina Szczotka [1,2,*,†], Shadi Nashashibi [3,†], Aleksandra Motyka [1,4], Sławomir Drozdek [1], Juerg Leuthold [3] and Karol Malecha [2]

[1] XTPL SA, Legnicka 48E, 54-202 Wrocław, Poland; aleksandra.motyka@xtpl.com (A.M.); slawomir.drozdek@xtpl.com (S.D.)
[2] Department of Microsystems, Faculty of Electronics, Photonics and Microsystems, Wrocław University of Science and Technology, wyb. S. Wyspiańskiego 27, 50-370 Wrocław, Poland; karol.malecha@pwr.edu.pl
[3] Institute of Electromagnetic Fields (IEF), ETH Zurich, 8092 Zurich, Switzerland; shadi.nashashibi@ief.ee.ethz.ch (S.N.); leuthold@ethz.ch (J.L.)
[4] Institute of Low Temperature and Structure Research, Polish Academy of Sciences, Okólna 2, 50-422 Wrocław, Poland
* Correspondence: nina.szczotka@xtpl.com
† These authors contributed equally to this work.

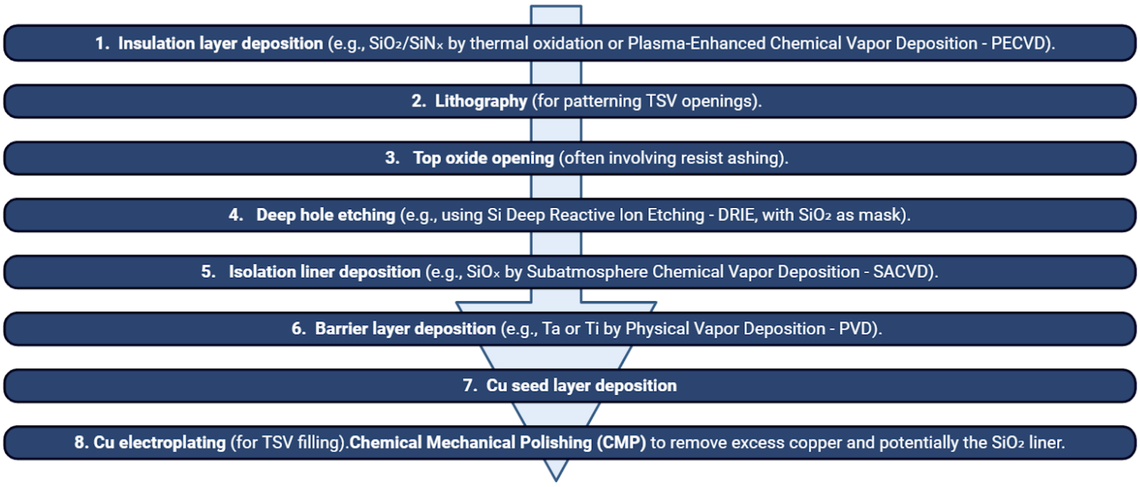

Figure S1. Scheme of common via filling process using the electroplating.

 https://doi.org/10.3390/ma19091861

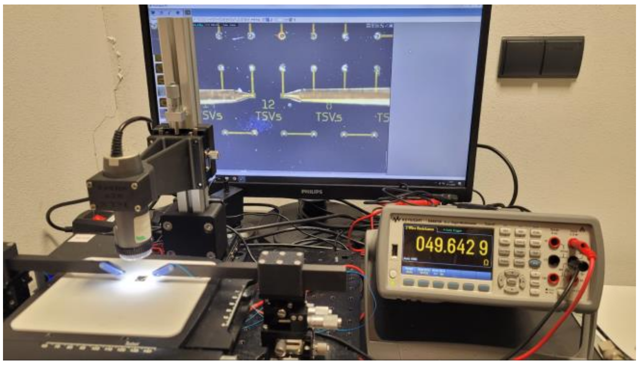

Figure S2. Electrical resistance measurement of a daisy chain test structure with 12 TSVs using a multimeter. A measurement station was constructed by XTPL specifically for the characterization of small-scale elements.

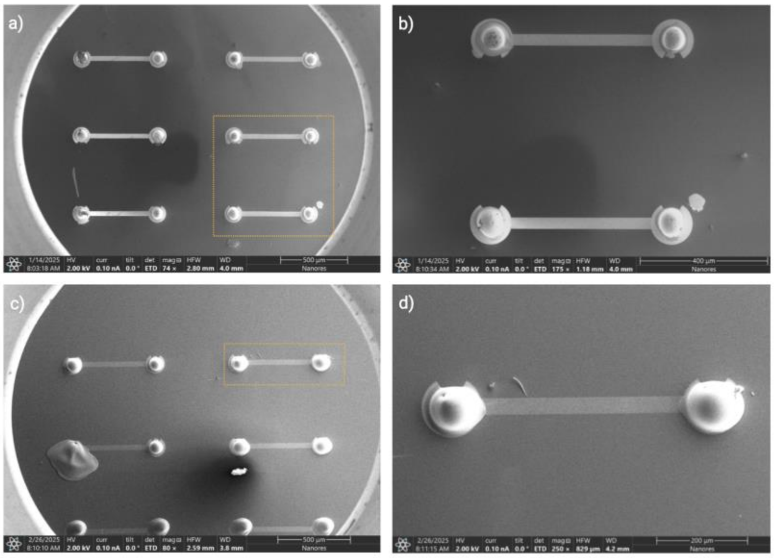

Figure S3. SEM images showcasing the morphology and filling quality of TSVs with a radius of 25 µm. Panels (a) and (c) present global top-down views, where orange frames highlight the regions magnified in panels (b) and (d), respectively. Panels (b) and (d) illustrate the excellent filling quality and consistent morphology of the silver bumps, with no visible cracks even under high magnification. Specifically, panel (b) shows two gold electrodes with four filled vias, while panel (d) focuses on a single electrode with two filled vias. These images confirm the successful and uniform filling of multiple vias within the daisy chain structures.

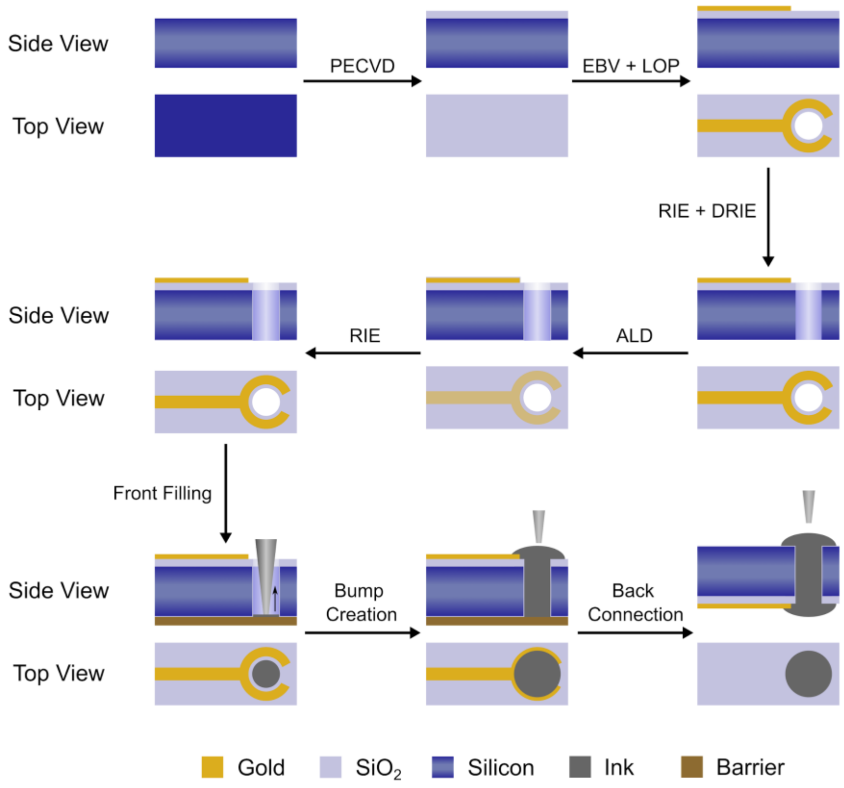

Figure S4. Full process flow from a silicon substrates to filled vias. Abbreviations: Plasma-Enhanced Chemical Vapor Deposition (PECVD), E-Beam Evaporation (EBE), Lift-Off Process (LOP), Reactive Ion Etching (RIE), Deep Reactive Ion Etching (DRIE), Atomic Layer Deposition (ALD).
